# Treatment as usual psychological therapy for complex post‐traumatic stress disorder in National Health Services in Scotland

**DOI:** 10.1111/bjc.70046

**Published:** 2026-03-13

**Authors:** Edel Mc Glanaghy, Catrin Lewis, Jonathan I. Bisson, Lewis Blair, Marylene Cloitre, Thanos Karatzias

**Affiliations:** ^1^ Adult Psychological Therapies NHS Forth Valley, Falkirk Community Hospital Falkirk UK; ^2^ School of Health and Social Care Edinburgh Napier University Edinburgh UK; ^3^ School of Medicine Cardiff University Cardiff UK; ^4^ Silver School of Social Work New York University New York New York USA

**Keywords:** clinical practice, complex PTSD, evidence base, psychological therapy, psychotherapy, treatment as usual

## Abstract

**Objectives:**

With Complex Post Traumatic Stress Disorder (CPTSD) as a new diagnostic category, there is a diversity of interventions and influences on clinical practice, it is prudent that we gather information about current ‘treatment as usual’. This study aims to address this need by describing the current clinical landscape of CPTSD treatments offered in NHS services in Scotland.

**Methods:**

An online survey was distributed via heads of service managers to clinicians working with adults. The survey posed questions about psychological therapy models, professional experience and, for the clinicians' latest 3 discharged cases, the length of therapy and perceived patient outcomes.

**Results:**

Forty‐nine clinicians, most of whom were clinical psychologists, took part and provided data about 139 clinical cases. Twenty‐three different therapeutic models were cited by clinicians, with phase‐based approaches described by 64% of clinicians. The modal number of sessions was 21–30. Most cases were described as completed, with 28% ending before therapy was completed, and a further 7% dropped out. Of those who completed therapy, 86% were judged to have improved after therapy, with 11% judged to have deteriorated.

**Conclusions:**

There is a wide variety of treatments available for complex PTSD, and innovative interventions and trials are required to support future clinical decision‐making about the optimal treatment components and therapy length.

**Practitioner points**

- CPTSD is a new diagnostic condition; however, clinical evidence is not yet available to direct treatment.
- This paper describes current practices in NHS psychological therapy services, in this vacuum across therapy modality and number of sessions provided.
- There is wide variation in clinical practice; however, most clinicians (89%) offer trauma memory reprocessing, and two‐thirds offer phase‐based approaches. Completed therapy involved 21–30 sessions.
- As new evidence emerges for what works best for CPTSD, this paper provides a benchmark of what services are currently offering.

## INTRODUCTION

Complex post‐traumatic stress disorder (CPTSD) was first introduced in the International Classification of Diseases, 11th Revision (ICD‐11) in 2018, a culmination of decades of research evidencing the developmental and wider mental health impacts of traumatic experiences. Meta‐analytic estimates indicate that up to 12.4% of people in nationally representative samples experience CPTSD, with clinical samples reporting a pooled prevalence of 44.7% (Huynh et al., 2025).

CPTSD includes the PTSD symptom clusters of re‐experiencing in the here and now, avoidance and a sense of threat plus three symptom clusters under the category ‘Disturbance of self‐organization’ (DSO): affect dysregulation, negative self‐concept and disturbed relationships (ICD‐11, 2018). Prior to the recognition of CPTSD as a specific condition, individuals presenting with these symptoms were likely diagnosed with multiple co‐morbid conditions such as PTSD, anxiety, depression, developmental trauma, dissociation, personality disorders and emotional dysregulation difficulties (Bryant, 2022; Maercker, 2021) and therapeutic approaches were adapted accordingly. In the UK, patients presenting with severe and enduring mental health difficulties, as well as patients presenting in crisis and with high levels of risk, are served by multi‐disciplinary teams where psychological intervention may be one element of a care package. Psychological interventions recommended by NICE (2018) for PTSD such as trauma‐focused cognitive behavioural therapy (TFCBT) and eye movement desensitization and reprocessing (EMDR) have been adapted for CPTSD based on studies with specific populations who have experienced complex trauma, such as childhood sexual abuse and military trauma (Karatzias et al., 2019), which has also informed clinical practice in the vacuum of a CPTSD evidence base.

Clinical practice and guidance (UKPTS, 2017) has historically been heavily influenced by Judith Herman's (1992) phase‐based approach, which emphasized a phase of safety and stabilization before addressing trauma memory re‐processing in phase two, and a less well established third phase, which focuses on future goals and post‐ recovery sense of self. Recent evidence for this phase‐based approach is mixed, with some RCTs finding that a phase‐based approach, while beneficial, may not be required, nor offer an advantage over PTSD‐focused therapy alone (e.g., Sele et al., 2023) however systematic review evidence indicates that there may be added benefit when considering outcomes other than PTSD symptoms (Darby et al., 2023), such as the DSO symptoms of CPTSD.

NHS psychological therapies services in Scotland are made of clinical and counselling psychologists, nurse psychotherapists, Clinical Associates (CAAPs) and other psychotherapists trained in psychological therapies as recommended by the Matrix—A guide to delivering evidence‐based psychological therapies in Scotland (NES Matrix, 2025). While there is a large overlap between clinical and counselling psychology, the doctoral training ethos differs slightly. According to the British Psychological Society (2025), clinical psychologists aim to ‘reduce distress’ within health systems, whereas counselling psychologists aim to improve psychological function. Clinical associates are psychologists trained to a master's level in a specific specialized area, such as CBT in adult primary care settings. Mental health nurses accredited in psychological therapy tend to adopt the ‘Nurse psychotherapist’ title.

Clinical and Counselling Psychologists are trained in multiple evidence‐based therapeutic approaches and are encouraged to apply interventions based on psychological formulation, rather than psychiatric diagnosis (Challoner & Papayianni, 2018). While this approach supports person‐centred working, in an evidence vacuum, such as there is for CPTSD, there is potential for individual therapists to create their own unique ‘package’ of therapy based on their modalities and preferences‐ possibly creating as many interventions as there are therapists. This can negatively impact on clinical governance and hamper patient care.

All mental health services have seen a rise in referrals and an increase in the number of complex presentations amongst these (Royal College of Psychiatrists, 2024), due in part, perhaps, to increased recognition of the impact of trauma and efforts to reduce stigma around help‐seeking in public spheres (NHS Education for Scotland, 2025). Service provision in primary care psychological services in England, that is, Talking Therapies, is well documented, yet there is little documentation on what generic NHS psychological therapy services are providing in the United Kingdom for CPTSD.

With CPTSD as a new diagnostic category there is an opportunity for the field to identify targeted and effective treatments which will require RCT evidence‐based trials. In situations of clinical equipoise, comparison against active control and/or current practice is preferred to avoid unethical comparisons with inactive treatments(Folefac & Desmond, 2024). Currently, best practice in the United Kingdom is defined as the use of evidence‐supported interventions; however, given the diversity of interventions this may yield and the wealth of influences on clinical practice, it is prudent that we gather information about current ‘treatment as usual’ (TAU). This study aims to address this need by describing the current clinical landscape of psychological therapies offered in the NHS for CPTSD, including psychological therapy models, therapy length and impact on patient outcomes. This information will allow for the characterization of TAU control groups in NHS settings and inform service training needs and governance as the evidence for effective therapies for CPTSD emerges.

## MATERIALS AND METHODS

### Procedure

Practicing psychological therapists, based in NHS psychological therapies services in Scotland were invited to complete an anonymous survey. The survey was open for 2 months, from 12th February 2024 to 12th April 2024.

### Setting and participants

All clinicians working in adult psychological therapies services in Scotland were eligible. According to official data, there were 1082 whole time equivalent staff employed in NHS services across Scotland (NHS Education for Scotland: Data, 2024). Respondents provided information on their job title, team and years of experience. They were also asked to describe, in anonymized form, their three most recent CPTSD cases (as described using ICD‐11 criteria). No demographic information was sought about these patients to strictly maintain patient anonymity.

### Data collection

A short survey was created on Microsoft Forms, based on therapy variables of interest, and reviewed by the authors, which include CPTSD clinical trial expertise.

The survey was distributed via MS Forms, with a link shared in an email via management networks; Heads of Psychological Therapies services and Heads of Adult Mental Health services in Scotland, to reach all staff working in NHS with CPTSD. A consent form preceded the survey questions explaining the purpose of the survey and how the data would be used. A copy of the survey is included in Appendix S1.

Completed survey responses were downloaded in an Excel sheet and any potentially identifiable information in qualitative responses was immediately anonymized. There was minimal missing data, with just one value missing. This was removed from the relevant analysis only.

### Data analysis

Quantitative data about the number of patient sessions and categorical data for clinician‐reported outcomes was coded and summarized using descriptive statistics. The number of sessions was summarized across each category of completion and by category of improvement. Clinician information was also summarized using descriptive statistics.

Quantitative data was analysed using Content Analysis (Erlingsson & Brysiewicz, 2017). The text responses about therapy models were coded and categorized by models and approaches listed. Triangulation involved the presentation of the results to staff teams across 2 different psychological therapy teams in different NHS health boards. At both meetings, the results were accepted as ‘validating’ by clinicians of their experience of service delivery.

#### Reflexivity statement

The lead author is a clinical psychologist, clinical supervisor and team lead in an NHS psychological therapies service in Scotland and a researcher in CPTSD. The author is trained in delivering CBT, Interpersonal Therapy (IPT) and EMDR and delivers formulation‐focused evidence‐based therapy to a clinical caseload. Compassion‐Focused Therapy (CFT), Schema therapy and integrative models of therapy are also delivered within the lead author's service. The project and coding were approached from a service delivery perspective, summarizing the therapeutic models used, the sequence of treatment and decision points for clinicians in applying their approach.

### Ethical approval

This project was designated as a service evaluation, capturing current practice and Caldicott approval was sought and received within NHS Forth Valley. As the survey was sent to colleagues across multiple health boards, the Public Benefit and Privacy Panel for Health and Social Care was consulted and approved the study.

## RESULTS

### Clinician characteristics

Forty‐nine participants completed the survey; however, two were removed as their professional role was identified as not being qualified (that is, an assistant and a trainee). The vast majority were applied psychologists. Most (64%) were clinical psychologists. The remaining clinicians were counselling psychologists (15%), nurse psychotherapists (11%) and other psychological therapists (10%).

**FIGURE 1:**
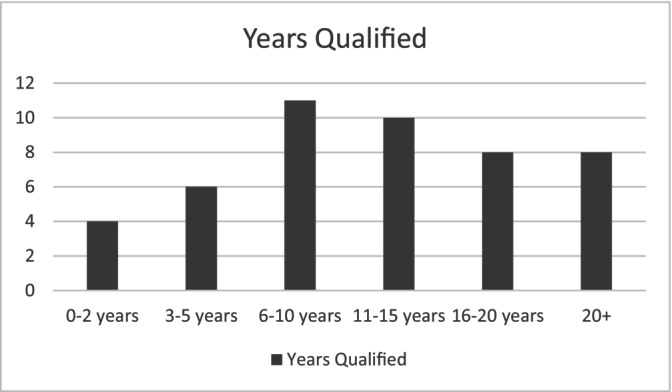
Number of years qualified as a professional therapist.

Most reported working in adult psychological therapy services (40%), secondary care psychology (32%) and older adult services (9%). Other specialities included learning disability services, clinical health psychology, eating disorders, prison psychological therapies, occupational health and substance use services. Most participants were qualified 6–10 years or 11–15 years, with representation across all levels of experience. Full details are presented in Figure 1.

### Therapeutic models and delivery

Of the 47 participants responding to the qualitative question ‘What do you typically provide for CPTSD?’, 30 (64%) mentioned safety and stabilization and/or used the term ‘phased based approach’, with five of these being clear that this was ‘as required’ and not offered to all patients. This work was often conflated with ‘emotional regulation’ in the text responses. Two participants indicated that safety and stabilization were ‘enough’ for some patients and that at times they do not need to offer further treatment for CPTSD symptoms. Most (89%) described using a memory reprocessing therapy in their responses (i.e., EMDR, TFCBT, PE and/or imaginal exposure). Full details are presented in Table 1. On the multiple choice list, 81% of the sample indicated that phase 1, safety and stabilization approaches were part of their ‘toolkit’.

**TABLE 1:** Psychological models and approaches used in treating CPTSD as indicated by clinicians.

Therapeutic approach	Chosen from list		Mentioned in text response	
	*n*	%	*n*	%
CBT with a trauma focus/TFCBT	38	81%	39	83%
Phase‐based safety & stabilization	38	81%	30	64%
Emotional regulation skills development	37	79%	24	51%
Compassion‐focused therapy	31	66%	16	34%
EMDR	26	55%	24	51%
Mindfulness	21	45%	0	0%
Prolonged exposure	17	36%	7	15%
Assertiveness skills	17	36%	2	4%
Acceptance & commitment therapy (ACT)	14	30%	6	13%
Dialectical behavioural therapy	14	30%	3	6%
Narrative exposure therapy	14	30%	2	4%
Schema therapy	11	23%	8	17%
Interpersonal therapy (IPT)	8	17%	2	4%
Supportive counselling	6	13%	0	0%
Mentalization‐based therapy	5	11%	0	0%
Cognitive analytic therapy (CAT)	2	4%	2	4%
Other: imagery rescripting			3	6%

*Note*: Other (*n* = 1 for each): Blue printing therapy, cognitive therapy, comprehensive resource model.

Abbreviation: EFT, psychodynamic therapy Qigong.

Forty participants (85%) referred to using TFCBT, CBT and/or Ehlers and Clarke (2000) model, while 24 (51%) referred to using EMDR. All but six of the clinicians cited TFCBT and/or EMDR. These six were described using other models such as Compassion‐Focused Therapy (*n* = 4), Schema Therapy (*n* = 2), psychodynamic therapy and Acceptance and Commitment Therapy. Many other therapeutic models were mentioned by the participants in the qualitative text box.

Compassion‐Focused Therapy (including compassionate mind training) was identified as highly popular, with more than half of participants (66%) using it in some capacity when choosing from the multiple‐choice list. It was also widely cited in the open‐ended text responses; however, it was applied in different ways, that is, by some as a phase 1 approach to develop skills for soothing, by others as a method of memory processing and by others for post‐processing work. Prolonged Exposure (35%) and Narrative Exposure Therapy (30%) were also frequently chosen from the multiple‐choice list. Assertiveness training and mindfulness were not mentioned in the qualitative text responses but were indicated as highly used from the multiple‐choice list.

Twenty participants (43%) specifically referred to using formulation to determine interventions. Two participants referred to assessing dissociation as part of their treatment plan specifically. Eleven participants (23%) of the full sample described using multiple models in a blended/integrative fashion, while 9 (19%) described applying interventions in a modular fashion, using a distinct specific approach for each specific difficulty. Three participants (6%) referred to phase 3 which involves reintegration, as part of their approach.

Most clinicians reported that sessions typically lasted 50/60 min, with just 3 reporting offering 30‐min sessions. Twenty‐five (53%) clarified that while they offer 50/60 min sessions, they also offered longer sessions of 90 min for trauma memory reprocessing.

### Therapist reported patient outcomes

The 47 clinicians reported outcomes for 139 patient cases. Most (79: 57%) indicated therapy was completed when ended, with 39 (28%) citing therapy was incomplete when sessions ended. 10 (7%) described premature ending due to dropout/DNA and 11 (8%) indicated other reasons, including eight that ended when the therapist moved, one patient moved from the area, one patient died and one reached their session limit.

**FIGURE 2:**
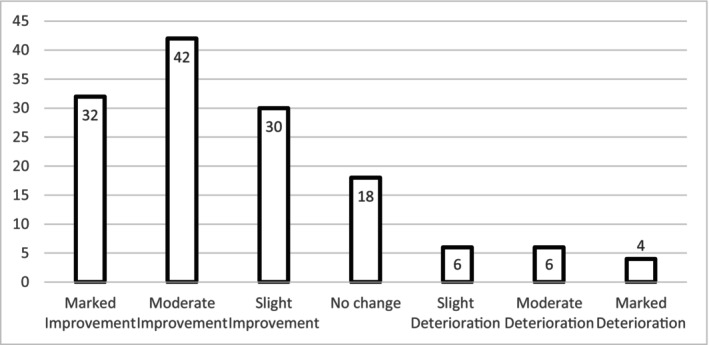
Perceived clinical outcome as indicated by clinicians.

Clinicians reported that the majority of patients (75%) had improved at the time of ending therapy: see Figure 2 for details, and Table 2 for a description of patient outcomes and categories of how therapy ended.

**TABLE 2:** How therapy ended, and the clinician described the outcome.

	Completed, *n* = 79 (100%)	Incomplete, *n* = 39 (100%)	DNA/dropout, *n* = 10 (100%)	Other, *n* = 11 (100%)
Improved (*n* = 104)	68 (86%)	24 (62%)	2 (20%)	10
No change (*n* = 18)	1 (1%)	11 (28%)	6 (60%)	0
Deteriorated(*n* = 16)	9 (11%)	4 (10%)	2 (20%)	1
	1 missing			

**FIGURE 3:**
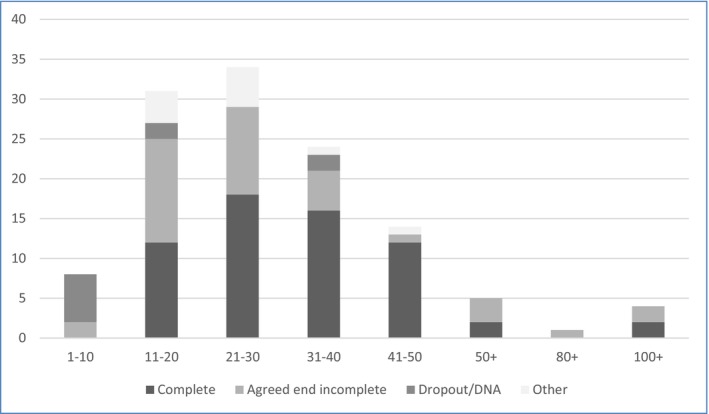
Number of therapy sessions and how therapy ended.

**FIGURE 4:**
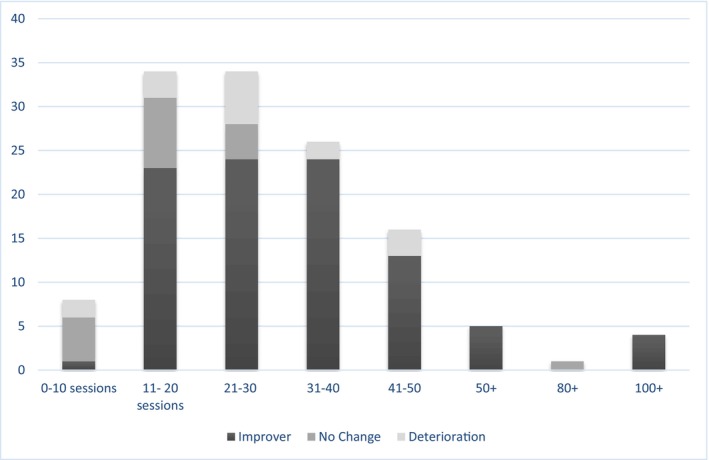
Number of sessions and perceived clinician‐rated patient outcome.

Clinicians indicated the number of sessions attended by each patient from a multiple‐choice list. All cases with 1–10 sessions were described as DNA/Dropout or Agreed End Incomplete. Completed cases involved between 11–20 and 100+ sessions. The most common number of sessions was 21–30. See Figure 3 for further details on the number of sessions and how therapy ended. Figure 4 depicts the number of sessions attended and the clinician‐described patient outcome.

## DISCUSSION

As ICD‐11 CPTSD is a new condition in clinical practice, it is important to explore the nature of psychological therapy that is currently being offered in statutory services. This information is essential for future clinical governance and hypothesis generation. The findings of this survey point to considerable variation in the clinical practice of clinicians working with adults who present to NHS services with CPTSD across Scotland. This variance was noted in the clinician's ‘typical’ plan for intervention with some citing ‘phased based’ approaches and others indicating more model‐specific approaches or formulation‐led intervention. There was also variance across the wide scope of psychological therapy models applied and number of sessions offered, with recognition that session limits were a feature for some services, but not others.

Clinician‐reported therapy completion rates were higher than expected, with 57% of cases reportedly completed, compared with a 33% completion rate in service data from a psychological therapies service (Brosnan & Mc Glanaghy, 2025). Clinician‐reported improvement was noted in 74% of cases, or 53% when considering only those described as showing at least moderate improvement. Best comparable evidence from real‐world settings, Talking Therapies 2023/24, indicate recovery rates for anxiety and depression at 50.1% (NHS Digital, 2024). CPTSD is arguably a more complex presentation than anxiety or depression alone, so the reported 53% here should be interpreted with caution. As this study only included clinician‐reported outcomes and relied on clinician's recall of cases, clinician bias may play a role. Future research would benefit from patient‐reported outcomes, using clinically robust measures.

The definition of dropout from psychological therapy varies across studies and trials, and therapy modality can be a factor (Penix‐Smith & Swift, 2025). The reported DNA/dropout rate of cases for this sample, as described by clinicians, was 7%; however, including therapy that ended prematurely, albeit with therapist agreement, would indicate 35% did not complete therapy. Dropout rates for PTSD clinical trials are reported to be 16% to 20% (Lewis et al., 2020; Varker et al., 2021), whereas rates in clinical practice range from 15% (Semmlinger et al., 2025) to 21% for people presenting for treatment for trauma‐related difficulties (Marsh et al., 2025). The data in this study, should be interpreted with caution as it relates to wide and varied clinical practice and is clinician reported. Further research is required to gather valid and comparable rates of dropout from psychological therapy for CPTSD across services and psychological therapy models.

Of those who were reported to have improved, the vast majority had completed therapy; however, 61% of those who did not complete therapy were also reported to have improved somewhat. Such improvement rates, albeit clinician rated, do raise questions about the mechanisms at play in the early stages of therapy. On a similar note, a larger number of sessions did not appear to increase the likelihood of improvement, with 20% of those receiving 41–50 sessions deteriorating, compared with 7% of those receiving 31–40 sessions deteriorating. Further data is required to understand the factors associated with deterioration, whether dose response has a role in this, and if there are clinical indicators when to cease therapy. The most commonly reported number of sessions was 21–30, understandably above the PTSD guidance of 8–12 sessions (NICE, 2018); however, in line with a retrospective study of trauma‐focused psychotherapy (namely EMDR or TF‐CBT) which reported an average of 28 sessions, with a range: 7–60 sessions (Melegkovits et al., 2022).

This survey summarizes a wide range of session lengths, ranging from 30 to 90 min sessions, with a consensus of 50/60 min sessions with optional 90 min sessions for trauma reprocessing sessions. This discrepancy can have implications for service delivery and resources required, particularly where caseloads include high numbers of CPTSD cases. Economic analysis of future RCTs for CPTSD treatments would benefit from consideration of session lengths for different therapy models.

Overall, there was a consensus that treatment for CPTSD requires an element of trauma memory reprocessing, with 89% of the therapists listing a processing element in their typical treatment plan. Furthermore, most continued to apply a phase‐based approach in their typical treatment, with just over a third not doing so. Although, this reflects an academic and clinical lack of consensus on whether phase‐based approaches are essential, beneficial or unnecessary and/or the legacy of the influence of phase‐based models, it also suggests that most therapists in clinical practice use a mixture of skills‐based interventions (i.e., emotional regulation) accompanied by trauma‐focused memory reprocessing work. This is consistent with the modular approach to therapy, such as ESTAIR (Karatzias et al., 2023) which has been advocated as a promising area of enquiry with some positive early findings (Karatzias et al., 2024).

Investigation into the factors that influence the implementation of research findings into clinical practice for psychological therapy highlights that supervision practice holds a more influential role than research findings (Gyani et al., 2014) and that treatment modifications away from the evidence base are common and based on clinician characteristics (Bruijniks et al., 2018). The findings must be considered in the context of this gap between research evidence and service delivery gaps as the evidence base for CPTSD emerges.

### Strengths and limitations

Any interpretation of this survey should consider the high likelihood of clinician bias in choosing which cases to describe and a lack of corroborating patient data. Beyond social desirability, it is possible that clinicians may have found it easier to recall more ‘extreme’ cases, that is, those who did well or those who did not, rather than recalling cases where progress was stalled or ended in an uneventful manner, such as being discharged due to access policies. Further, as this survey was distributed by service managers, there may have been a service selection bias as well. It is also possible that the sample, which was a small proportion of the full population of professionals delivering psychological therapy, was not representative, however presentations to 2 distinct team did affirm this study's findings.

Another key limitation is the lack of information about clinician assessment tools for CPTSD, which impacts the robustness of the clinical case sample. Furthermore, using clinician‐reported patient outcomes and information regarding attrition/lack of engagement could have led to biased reporting. Future studies would therefore benefit from inclusion of patient reported outcomes. This is especially important in relation to those who were described as deteriorating to understand if therapy had an adverse effect (Parry et al., 2016).

Furthermore, the sample included professionals across a range of clinical experience and service types, all of which may have interacted with therapy delivery, limiting generalizability. Despite this, this study provides some initial insight into TAU in clinical services and reflects a coherent picture of both skills‐based and trauma memory reprocessing elements being utilized. The inclusion of a self‐report text box, followed by a multiple‐choice list of therapy options, was a strength of this paper. It allowed clinicians to describe their ‘typical’ practice and then prompted for other models they use allowing for the inclusion of practices that may have been overlooked with just one data type. While this study is purely descriptive, it may provide a benchmark for other services against current practice and indeed may serve as a baseline for improvement work to ensure vital resources are delivered as efficiently as possible within NHS services.

Strengths of this study include its timely focus on a relatively newly introduced clinical diagnosis, its national scope and the inclusion of clinicians from a range of experience levels. By collecting real‐world data from across NHS psychological therapy services in Scotland, this survey provides valuable insight into current treatment practices for CPTSD. The inclusion of data from the most recent cases also helps reduce retrospective bias and offers a snapshot of care being delivered on the ground.

## CONCLUSION

These findings demonstrate that combining more than one model of therapy when working with CPTSD is the norm rather than the exception in real‐world clinical practice. Further research would be helpful on how, when and why therapists choose to combine or change models within an integrated or multi‐model therapeutic approach, considering the role of formulation as well as the impact of the emotive nature of trauma memory reprocessing on therapists. Guidelines such as The Matrix, and NICE, could support best practice by including guidance not just on the effectiveness of individual models of therapy but also on models of integration (e.g., sequential, modular, etc.). Further research is required to gather valid and comparable rates of engagement and recovery across services, including patient reported and clinically robust measures.

## AUTHOR CONTRIBUTIONS

**Edel Mc Glanaghy:** Conceptualization; investigation; methodology; visualization; formal analysis; project administration. **Catrin Lewis:** Conceptualization; methodology; writing – original draft. **Jonathan I. Bisson:** Conceptualization; methodology; supervision; writing – original draft. **Lewis Blair:** Investigation; writing – review and editing. **Marylene Cloitre:** Conceptualization; writing – review and editing; supervision. **Thanos Karatzias:** Conceptualization; writing – original draft; supervision.

## CONFLICT OF INTEREST STATEMENT

There are no financial conflicts of interest.

## Supporting information


Appendix S1

## Data Availability

Raw data will not be made available, as per NHS Forth Valley Caldicott permissions.
