# Supplementary material for: Treatment as usual psychological therapy for complex post‐traumatic stress disorder in National Health Services in Scotland
Source: Br J Clin Psychol. 2026 Mar 13;65(2):708–19. doi: 10.1111/bjc.70046 (PMC13159763; doi:10.1111/bjc.70046)
Supplement: Supplementary file 1 — Appendix S1 [file BJC-65-708-s001.docx]

**Treatment as Usual psychological therapy for Complex Post Traumatic Stress Disorder in National Health Services in Scotland**

**Short running title: Treatment as usual for Complex PTSD in the NHS**

Appendix 1: Survey

Treatment as usual for Complex PTSD

This survey is for clinicians in the NHS providing psychological therapy for people presenting with complex PTSD as their primary difficulty. Complex PTSD involves PTSD symptoms plus emotional dysregulation, interpersonal difficulties and negative sense of self. This survey is anonymous and asks for information about your qualifications and what you offer to people presenting with Complex PTSD in general. We will then ask about your 3 most recent relevant CPTSD cases.

This information will help us to understand what treatment as usual is for CPTSD, and may inform the development of guidelines, highlight areas of practice, and support research trials that include a treatment as usual category for CPTSD.

If you have any questions please contact X.

1. What is your professional qualification (ie, the one under which you deliver psychological therapy)?

Clinical Associate Applied Psychologist/ IAPT therapist

Clinical Psychologist

Counselling Psychologist

Medical psychotherapist

Nurse psychotherapist

Psychiatrist

Psychotherapist

Other

2. How long have you been working in a therapy related service with people experiencing difficulties associated with Complex PTSD/trauma?

0-2 years

3-5 years

6-10 years

11-15 years

16-20 years

20 years +

3. What NHS service do you currently work for?

Adult Psychological therapies

CMHT/Secondary Care psychology service

Substance Use

Clinical Health psychology

Eating Disorders

Older Adult services

LD services

CAMHS

Other

4. Where is your NHS service based?

Scotland

England

Wales

5. Consider that you are assigned a case today where the patient meets criteria for Complex PTSD (PTSD symptoms plus emotional dysregulation, interpersonal difficulties and negative sense of self). Assuming this is the primary issue and there are no immediate risk concerns that require crisis input, can you give a brief description of what you would typically plan to offer as part of individual therapy?

This description may include specific therapy models, therapeutic components or procedures/protocols you tend to follow.

6. Please tick which aspects of therapy you typically include in your work with people with CPTSD- please choose all that apply, including standard service offers that your patients tend to have also engaged with.

Please note this list is not exhaustive and is not indicative of support for any approach.

Acceptance and Commitment Therapy (ACT)

Assertiveness skills

CBT with a Trauma focus/TFCBT

Cognitive Analytic Therapy (CAT)

Compassion Focused Therapy (CFT)

Compassionate mind skills

Dialectical Behavioural Therapy (DBT)

EMDR

Emotional regulation skills development

Interpersonal Therapy (IPT)

Mentalization based therapy

Mindfulness

Narrative exposure therapy

Phase based Safety & Stabilisation

Prolonged Exposure

Schema Therapy

Supportive Counselling

Other

7. How long are your therapy sessions (in minutes)? If there is variation, please describe.

8. Think back to the 3 most recent patients with CPTSD whom you worked with and are

NO LONGER SEEING.

Roughly how many therapy sessions were attended by Patient 1?

1-10

11-20

21-30

31-40

41-50

Other

How did therapy end for Patient 1?

Therapy completed

Agreed ending despite therapy goals not yet reached

DNA/Dropout

Other

In your opinion, when therapy ended, what was the status of Patient 1's Complex PTSD difficulties?

Marked Deterioration

Moderate Deterioration

Slight Deterioration

No Change

Slight Improvement

Moderate Improvement

Marked Improvement

*Question 8 repeated for Patient 2 and Patient 3.*

9. Is there anything else you want to tell us about how you provide treatment for people with Complex PTSD? This may include service level factors (such as session limits), training needs or other considerations. Please do not include any identifiable information.
